# Design of a nutrient solution supply device for a vertical aeroponic cultivation system

**DOI:** 10.3389/fpls.2025.1615927

**Published:** 2025-07-03

**Authors:** Yihan He, Xinghua Zhang, Ting Wang, Xinyu Tan, Shuguang Liu

**Affiliations:** Yantai Institute, China Agricultural University, Yantai, China

**Keywords:** aeroponics, nutrient solution, nutrient solution supply device, vertical farming, soilless cultivation, facility horticulture

## Abstract

Aeroponic cultivation systems provide precise environmental control for plant growth, effectively block the reproductive pathways of root diseases and pests, and facilitate efficient water resource recycling, thereby offering a scalable technical solution for intensive, high-yield, and sustainable agricultural production. Aiming to address the complex underground pipelines and excessive spray nozzles in fixed-pipeline nutrient solution supply systems of conventional vertical aeroponic cultivation, this study proposes a vertical aeroponic cultivation paradigm for large-scale production that can be applied to mobile nutrient solution supply modes. This study analyzes the structural components and operational workflow of the vertical aeroponic cultivation system, and the structural components, operational principles and technical specifications of the dedicated nutrient solution supply device. Transient dynamic analysis is conducted using ANSYS Workbench 2025 R1 (student) software, thereby yielding the equivalent stress/strain distributions on the body frame at 0.15 m/s, and on the spray bracket at 0.4 m/s. The simulation results demonstrate that both structures maintain stress within material limits and without significant concentration areas, with minimal strain levels meeting operational requirements. The Box-Behnken experimental design methodology is adopted to establish flow rate of the spray nozzles, moving speed of the spray nozzles, and vertical height from the test points on the legs of the trapezoidal cross-section of the cultivation bed to the ground as experimental factors, with nutrient solution coverage rate as evaluation indicator. The experimental results are analyzed using Design Expert 13.0 software to establish a regression model and conduct optimization analysis. The optimization results indicate that with a flow rate of 3 L/min for the spray nozzles and a moving speed of 0.38 m/s for the spray nozzles, the nutrient solution coverage rates on the legs of the trapezoidal cross-section of the cultivation bed reach >90% for both vertical height positions (0.1 m and 1.4 m from ground). This configuration ensures >90% nutrient solution coverage rate across the entire legs of the trapezoidal cross-section of the cultivation bed. Verification experiments confirm nutrient solution coverage rates at the respective test points are 90.33% and 91.52%, which is in accordance with the practical application requirements. This study will serve to diversify aeroponic production methodologies, expand development potential for commercial aeroponics, and provide valuable insights for technological dissemination.

## 1 Introduction

Aeroponic cultivation, a revolutionary approach, exposes plant roots to air and delivers essential nutrients through misting (Garzón et al., 2023). This method simultaneously satisfies plants’ requirements for water, nutrients, and root zone oxygenation (Lakhiar et al., 2018), demonstrating significant agronomic advantages (Lian et al., 2020). By resolving the critical water-oxygen balance challenge in root zone management (Liu et al., 2017), aeroponics maintains roots in an optimally controlled environment through spraying nutrient solution delivery, ensuring precise moisture regulation and aeration (Salazar et al., 2020). This technique eliminates risks from soil-borne pathogens and aquatic pests (Yu et al., 2019), thereby unlocking plants’ full growth potential and improving both crop quality and yield (Thakur et al., 2019; Tokunaga et al., 2020). With demonstrated benefits in land-use efficiency (Giurgiu et al., 2017), root microenvironment control (Li et al., 2018; Lakhiar et al., 2019; Yang et al., 2022), and biosecurity (Lakhiar et al., 2020), aeroponics represents a transformative solution for sustainable agriculture (Qi et al., 2022). Currently, the theoretical progress of aeroponics has been widely acknowledged.

In recent years, aeroponic research has predominantly focused on three interconnected domains: optimization of core components, intelligent monitoring and control systems, and nutrient solution management. Studies on core component optimization have delved into the design and performance of atomization nozzles, a critical factor in nutrient delivery efficiency. Lawrance et al. (2025) employed computational fluid dynamics (CFD) to analyze how nozzle diameter, angular orientation, and Reynolds number influence spray flow characteristics, such as velocity distribution and liquid fraction development. Feng et al. (2024) developed a novel atomization nozzle, revealing the impacts of pressure and outlet diameter on key atomization parameters. Nguyen et al. (2023), established a predictive model for volumetric droplet size distribution (VDSD) in flat fan sprays using energy conservation principles. Tunio et al. (2021) investigated how droplet size and spraying intervals from different nozzles affected lettuce growth. Abbasi et al. (2024) optimized nutrient delivery systems (aeroponic and ultrasonic with varied pulse periods) for cut flower production. Mirzabe et al. (2023) explored piezoelectric atomization, analyzing factors like duty cycle frequency (DCF) and high-speed pulse current (HSPC) on misting rates.

In the realm of intelligent monitoring and control, researchers have increasingly integrated IoT, AI, and sensor technologies to enhance aeroponic system precision. Qureshi et al. (2025) explored the integration of plasma-activated water/mist with AI and IoT to address resource and nutrient management challenges. Dhanasekar (2025) reviewed IoT applications in smart agriculture, emphasizing their role in soilless cultivation (particularly aeroponics) for real-time monitoring of plant growth and nutrient absorption. Sadek et al. (2024) designed an IoT-based smart greenhouse system with multi-sensor integration for automated environmental control and remote monitoring, demonstrating its effectiveness in lettuce cultivation. Elsherbiny et al. (2024) developed a meta-learning framework using multimodal data (i.e. spectral, thermal, and IoT environmental data) to noninvasively monitor key physiological parameters (leaf relative humidity, chlorophyll, and nitrogen content) in real time. Chowdhury et al. (2021) focused on nutrient solution recirculation via ion-selective electrodes (ISEs), optimizing nutrient efficiency and reducing environmental pollution through sensor-based feedback.

Nutrient solution management research has centered on maximizing nutrient delivery efficiency, enhancing plant growth, and optimizing nutrient utilization. Liu et al. (2025) studied the effects of three-cycle nutrient recycling in a deep flow hydroponic system, revealing imbalances in nutrient uptake. Sereshkeh et al. (2024) introduced Staticaponics, a hybrid system combining electro-solution dynamics to enhance nutrient efficiency beyond traditional hydroponics or aeroponics. Al Farqani et al. (2024) investigated the influence of solution pH (5.5, 6.5, 8.0) on apple rootstock development in aeroponics. Grzelka et al. (2023) used aeroponics to study electroporation-induced stress on Scutellaria baicalensis growth and flavonoid production. Collectively, these studies highlight the critical role of dynamic nutrient adjustment, recycling strategies, and pH management in optimizing aeroponic productivity and sustainability.

Currently, fixed-pipeline nutrient solution supply systems in vertical aeroponic cultivation are characterized by complex underground pipeline networks and a high density of supporting spray nozzles. In this paper, we propose a mobile nutrient solution supply mode for vertical aeroponic cultivation and develop a dedicated mobile nutrient supply device for large-scale production. Using one mobile nutrient solution supply unit of the mobile nutrient solution supply mode to replace multiple fixed nutrient solution supply unit combinations of the fixed-pipeline nutrient solution supply mode eliminates the need for underground fixed nutrient solution supply pipelines and reduces the number of spray nozzles required. Thereby, this study reduces capital investment, diversifies aeroponic production methodologies, expands the development potential of commercial aeroponics, and provides valuable insights for technological dissemination.

## 2 Materials and methods

### 2.1 Overall structure and operational workflow of the vertical aeroponic cultivation system

#### 2.1.1 Overall structure

**Figure 1:**
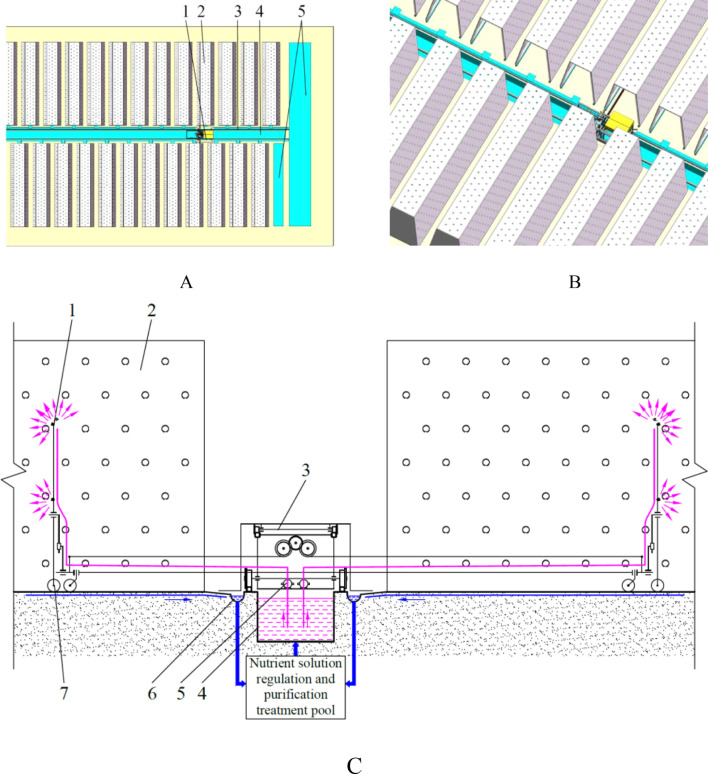
Overall structure and operational workflow. **(A)** plane view. 1. nutrient solution supply device 2. cultivation bed 3. nutrient solution return pipeline 4. nutrient solution supply pool 5. nutrient solution regulation and purification treatment pool; **(B)** aerial view; **(C)** operational workflow. 1. spray nozzle 2. cultivation bed 3. body frame of the nutrient solution supply device 4. nutrient solution supply pool 5. water pump 6. nutrient solution return pipeline 7. spray bracket.

The overall structure is made up of several key components, including a nutrient solution supply pool, cultivation beds, a nutrient solution supply device, a nutrient solution return pipeline, and nutrient solution regulation and purification treatment pools. The structural layout is depicted in **Figure 1**. The nutrient solution supply pool is characterized by a narrow and elongated configuration, extending along the length of the greenhouse. Its primary function is to store the nutrient solution required for plant growth. The cultivation beds, which function as the carrier for the vertical cultivation of plants, share the same length, within which plant roots absorb the nutrient solution and grow. To ensure unobstructed movement of the push-pull rod between the cultivation beds, the cultivation beds on either side of the nutrient solution supply pool are arranged in a staggered manner, with the longitudinal projections of both sides of the cultivation beds overlapping. The nutrient solution supply device is responsible for the application of the nutrient solution to the plant roots during the transfer process within the internal spaces of the cultivation beds. The nutrient solution return pipeline is distributed on both sides of the nutrient solution supply pool and extends along it. This pipeline functions as the conduit through which excess nutrient solution is directed into the nutrient solution regulation and purification treatment pools. The nutrient solution regulation and purification treatment pools are positioned at both ends of the greenhouse. These pools are responsible for the preparation and supplementation of the nutrient solution. Concurrently, the nutrient solution can undergo filtration and sterilization processes to ensure its quality conforms to the criteria necessary for optimal plant growth.

#### 2.1.2 Operational workflow

The operational workflow is illustrated in **Figure 1**. During operation, the water pumps of the nutrient solution supply device continuously draw nutrient solution from the nutrient solution supply pool. The position of the nutrient solution supply device and the position of the spray bracket, which is driven by the push-pull rod, are changed accordingly. The result is that the nutrient solution is evenly sprayed onto the plant roots inside the cultivation beds, thereby meeting the nutrient solution absorption needs of the roots. A proportion of the nutrient solution that has been sprayed onto the inner wall of the cultivation beds will subsequently run down the inner walls to the floor or drip directly to the floor beneath the cultivation beds. There is also a portion of the nutrient solution that is sprayed onto the root system but is not absorbed by the root system and cannot accumulate on the root system that flows down the root system to the end of the root system and then drips to the floor beneath the cultivation beds. The nutrient solution on the floor flows into the nutrient solution return pipeline. The refluxed nutrient solution is finally collected in the nutrient solution regulation and purification treatment pool. Following a series of treatment processes, including filtration and disinfection, the solution is discharged into the nutrient solution supply pool. This process establishes a nutrient solution recycling system, achieving efficient resource utilization.

### 2.2 Structure and working principle of the nutrient solution supply device

#### 2.2.1 Overall structure

**Figure 2:**
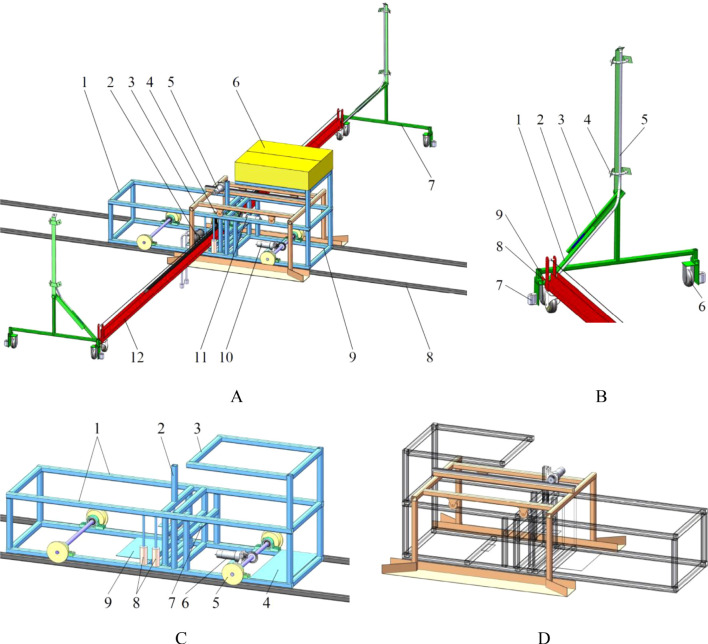
Nutrient solution supply device. **(A)** overall structure. 1. body frame 2. water pump 3. spray bracket transfer platform 4. winding roller 5. drive motor for the spray bracket transfer platform 6. control box 7. spray bracket 8. track 9. battery box 10. drive motor for the body frame 11. drive motor for the push-pull rod 12. push-pull rod; **(B)** spray bracket. 1. connecting rod 2. slider 3. guide rail 4. spray nozzle 5. transmission pipeline of nutrient solution 6. walking wheel of spray bracket 7. protection wheel 8. contact point between push-pull rod and spray bracket 9. rotating vice connection point between push-pull rod and spray bracket; **(C)** body frame. 1. track of spray bracket transfer platform 2. fixed position of the drive motor for the spray bracket transfer platform 3. fixed position of the control box 4. fixed position of the battery box 5. walking wheel 6. fixed position of the drive motor for the body frame 7. fixed position of the drive motor for the push-pull rod 8. contact roller 9.fixed position of the water pumps; **(D)** spray bracket transfer platform.

The structure of the dedicated nutrient solution supply device, as shown in **Figure 2**, includes a power system, a control system, a body frame, a spray bracket along with its push-pull rod, a spray bracket transfer platform, a nutrient solution spraying system, etc.

#### 2.2.2 Working principle

The Programmable Logic Controller (PLC), as the core of the control system, is responsible for regulating the start, stop, forward, and reverse operations of each drive motor, a function facilitated by the reception of signals from sensors. Subsequent to this, the PLC directs the execution structure to commence its designated function.

The body frame structure, which functions as the primary load-bearing components, provides support for both the functional and execution structures. The vehicle body is able to move in both the anterior and posterior directions along the predetermined trajectory. During this movement, the push-pull rod is driven to move perpendicular to its own axis, and the nutrient solution supply remains in a non-supplying state.

The spray bracket is attached to each extremity of the push-pull rod. In the absence of movement in the frame, the push-pull rod moves along its axial direction. The movement of one end of the push-pull rod is such that it exerts a forward force on a spray bracket, whilst the other end pulls the other spray bracket. When the spray bracket at the pushing end reaches its maximum displacement within the cultivation bed, the spray bracket at the pulling end simultaneously arrives at the spray bracket transfer platform. This results in the position of the spray brackets within the same cultivation bed being altered.

The spray nozzles are installed on the spray bracket. As the push-pull rod moves within the cultivation bed, the spray nozzles continuously spray, thereby providing the plant roots in the cultivation bed with a continuous supply of nutrient solution.

The spray bracket transfer platform is supported by the body frame and uses the body frame as a track. It is capable of performing relative displacement in the direction of the body frame’s movement. The transfer platform is oriented in the direction of the spray bracket at the pulling end of the push-pull rod. Upon the spray bracket at the pulling end reaching the transfer platform, the push-pull rod halts its movement. Consequently, the spray bracket on the transfer platform is driven towards the near port of the spraying space of the subsequent cultivation bed, thereby completing the position transfer of the spray bracket between different cultivation beds.

The nutrient solution contained within the nutrient solution supply pool is pumped out by water pumps. The solution is then conveyed through the conveying pipeline to the spray nozzles on the spray brackets, thereby achieving the supply of the nutrient solution in a spray form.

#### 2.2.3 Main technical parameters

The main technical parameters are shown in **Table 1**. The maximum dimensions of the body frame in the length, width, and height directions are 1630 mm, 608 mm, and 640 mm, respectively. The spray bracket is triangular in structure, with a bottom side length of 807 mm, a width of 86 mm, and a height determined by the nozzle arrangement position, which is 1150 mm in this design. The total length of the push-pull rod in this design is 6 m, suitable for greenhouses with a span of ≤15 m. The device’s motion speed parameters mainly include the body frame movement speed, the spray bracket movement speed, and the spray bracket transfer platform movement speed. The body frame movement speed is preferably ≤0.15 m/s due to the significant length of the push-pull rod, which may affect mechanical stability. The movement of the spray bracket is characterized by a velocity of 0.4 m/s, with the capacity to undergo adjustment in accordance with the demands of the plant nutrient solution, the flow rate of the spray nozzles, and the pressure of the water pumps. The movement of the spray bracket transfer platform at a velocity of 0.2 m/s ensures that the transfer platform reaches the designated position ahead of the body frame, thereby enhancing the efficiency of time utilization.

**Table 1:** Main technical parameters.

Main parameters	Unit	Value
Overall dimensions of the body frame (L_1_×W_1_×H_1_)	mm	1630*608*640
Dimensions of the spray bracket (L_2_×W_2_×H_2_)	mm	807*86*1150
Push-pull rod length	m	6
Moving speed of the spray bracket	m/s	0.4
Moving speed of the body frame	m/s	0.15
Moving speed of the spray bracket transfer platform	m/s	0.2

### 2.3 Main structure

#### 2.3.1 Power system

The power system is composed of drive motor for the body frame (Motor 1), drive motor for the spray bracket transfer platform (Motor 2), drive motor for the push-pull rod (Motor 3), and two water pumps (Water pump 1-2). In the course of power system operation, the drive motors do not function concurrently. When the body frame moves at a speed of 0.15m/s, the push-pull rod moves at a speed of 0.4m/s, and the spray bracket transfer platform moves at a speed of 0.2m/s, the power consumption of the nutrient solution supply system is 108 W. Given that the length of the cultivation beds is 5.5m, the length of the lower base of the cultivation bed cross-section is 1m, and the interval between cultivation beds is 0.4m, the working efficiency of the nutrient solution supply device is 1509m²/h.

#### 2.3.2 Control system

The control system encompasses a PLC, limit sensors, a proximity sensor, and other components. The specific PLC employed in this study is the Siemens PLC S7-200SMART ST40 controller. The configurations of these sensors are presented in **Table 2**.

**Table 2:** Sensors.

No.	Sensor Name	Type	Function
1	Sensor 1	Proximity Sensor	Start and stop of the frame structure
2	Sensor 2	Limit Sensor	Limit the motion of the spray bracket transfer platform along direction >②
3	Sensor 3	Limit Sensor	Limit the motion of the spray bracket transfer platform in the ② opposite direction
4	Sensor 4	Limit Sensor	Limit the motion of the push-pull rod along the direction ①
5	Sensor 5	Limit Sensor	Limit the motion of the push-pull rod in the ① opposite direction
6	Sensor 6	Limit Sensor	Limit the motion of the nutrient solution supply device along the direction ②
7	Sensor 7	Limit Sensor	Limit the motion of the nutrient solution supply device in the ② opposite direction

**Figure 3:**
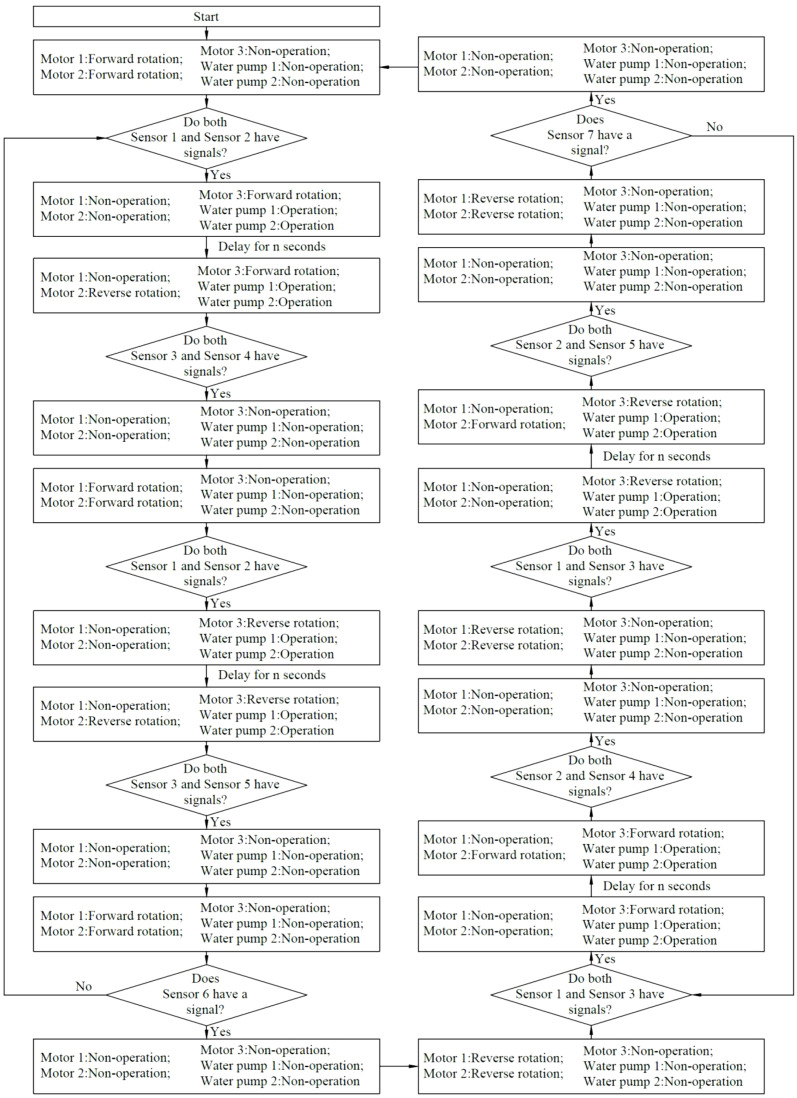
Control flow.

**Figure 4:**
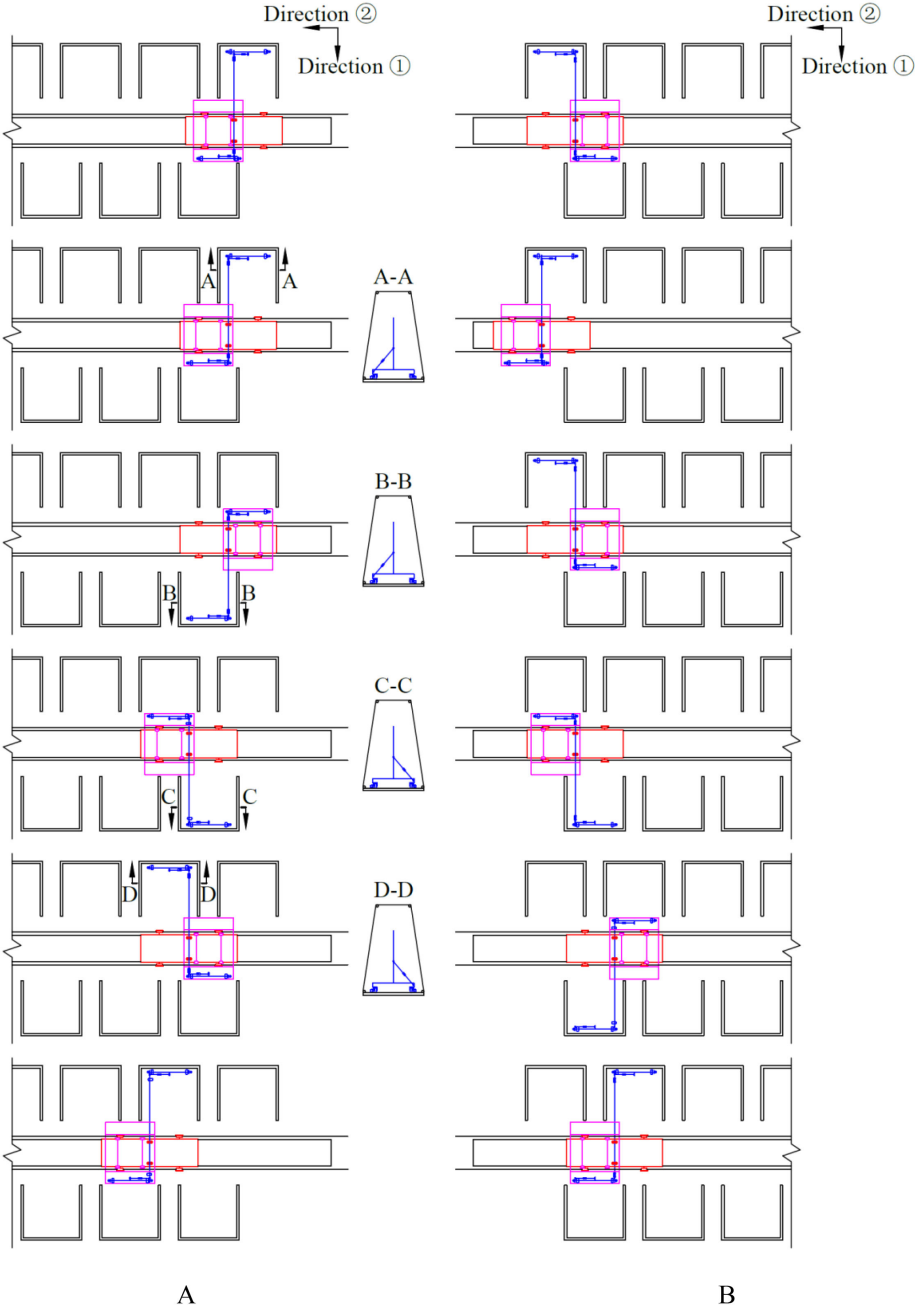
Detailed actions during the forward and backward movement process. **(A)** detailed actions during the forward movement process; **(B)** detailed actions during the backward movement process.

The control flow is illustrated in **Figure 3**. The control system governs the operation of the drive motors through the signals fed back by the sensors, thereby enabling the nutrient solution supply device to move in both forward and reverse directions. During this process, the actions of all the execution components collaborate with each other. The detailed actions involved in the forward and backward movement processes are illustrated in **Figure 4**. The red line denotes the body frame, the magenta line signifies the spray bracket transfer platform, and the blue line designates the spray bracket and the push-pull rod.

#### 2.3.3 Body frame

The body frame, which is composed of 30×30 steel pipes that have been welded together, is demonstrated in **Figure 2**. The dimensions of the designed body frame are determined as in Equation 1.


(1) $$\left\{ \begin{array}{l} W_{1}=(1.1-1.2)B_{1}\\ L_{1}=2L_{3} \end{array}\right.$$

Where, *W*_1_ is the width of the body frame, m; *B*_1_ is the width of the nutrient solution supply pool, m; *L*_1_ is the length of the body frame, m; *L*_3_ is the length of the spray bracket transfer platform, m.

The process of movement of the body frame is a process of non-nutrient solution supply. The velocity at which the body moves affects the efficiency of the work, and its walking speed is calculated as in Equation 2.


(2) $$v_{1}=\frac{\pi d_{1}n_{1}}{6\times 10^{4}i_{1}}$$

Where, *v*_1_ is the moving speed of the body frame, m/s; *d*_1_ is the diameter of the walking wheels, mm; *n*_1_ is the rotational speed of the drive motor for the body frame, r/min; *i*_1_ is the transmission ratio of the master-slave wheel of the bevel gear.

**Figure 5:**
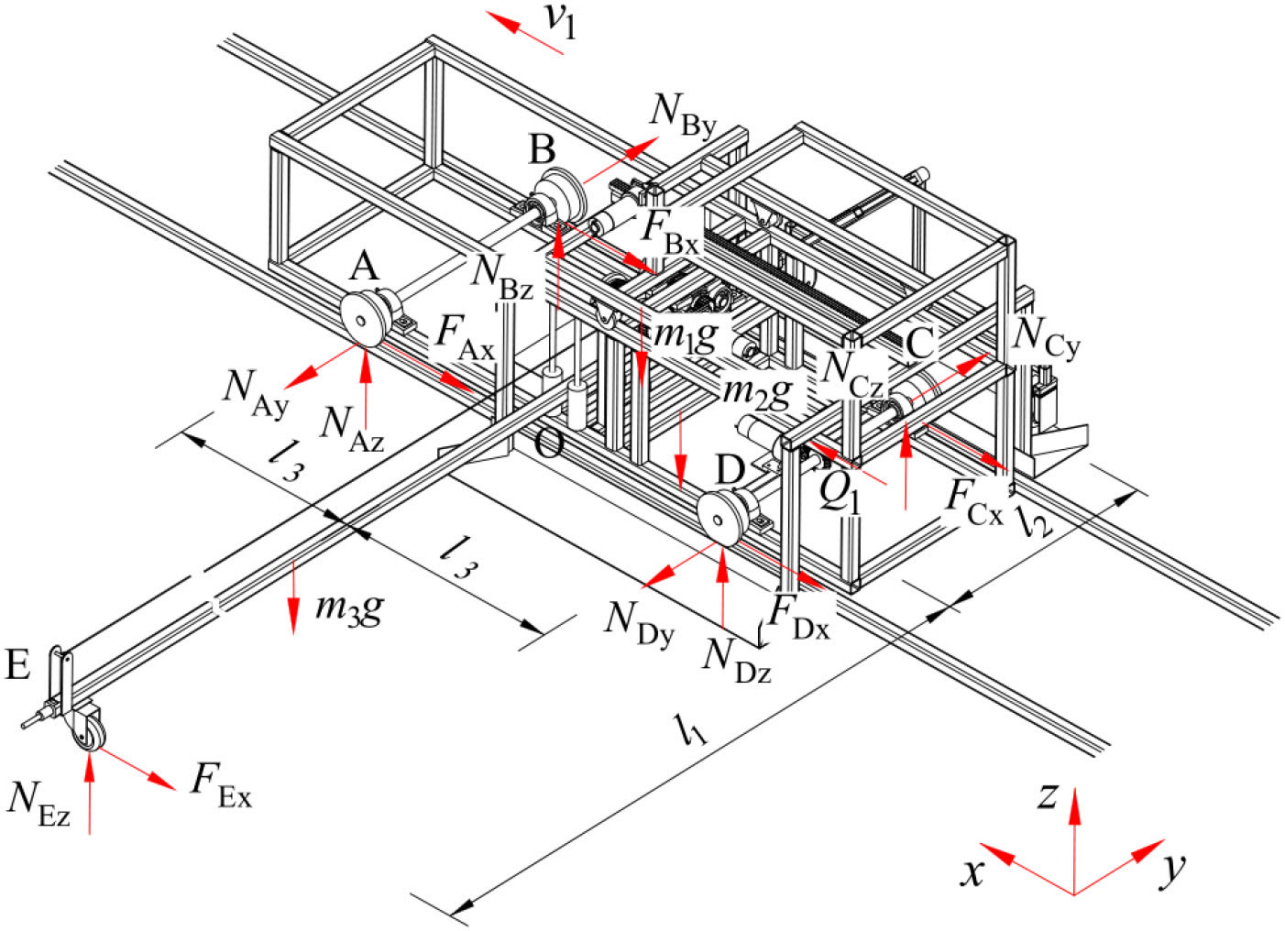
Force-bearing condition of the device.

When the device is operating stably, its force-bearing condition of the device is shown in **Figure 5**, and the mechanical relationship is expressed by Equation 3.


(3) $$\left\{ \begin{array}{l} Q_{1}-(F_{\text{Ax}}+F_{\text{Bx}}+F_{\text{Cx}}+F_{\text{Dx}})-F_{\text{Ex}}=0\\ N_{\text{Ay}}-N_{\text{By}}-N_{\text{Cy}}+N_{\text{Dy}}=0\\ N_{\text{Az}}+N_{\text{Bz}}+N_{\text{Cz}}+N_{\text{Dz}}+N_{\text{Ez}}-(m_{1}+m_{2}+m_{3})g=0\\ (N_{\text{Ay}}-N_{\text{By}}+N_{\text{Cy}}-N_{\text{Dy}})\cdot l_{3}+(\frac{Q_{1}}{2}-F_{\text{Bx}}-F_{\text{Cx}})\cdot l_{2}+F_{\text{Ex}}\cdot l_{1}=0\\ F_{\text{Ax}}=f_{1}N_{\text{Ay}}+\frac{\delta_{1}}{r_{1}}N_{\text{Az}}\\ F_{\text{Bx}}=f_{1}N_{\text{By}}+\frac{\delta_{1}}{r_{1}}N_{\text{Bz}}\\ F_{\text{Cx}}=f_{1}N_{\text{Cy}}+\frac{\delta_{1}}{r_{1}}N_{\text{Cz}}\\ F_{\text{Dx}}=f_{1}N_{\text{Dy}}+\frac{\delta_{1}}{r_{1}}N_{\text{Dz}}\\ F_{\text{Ex}}=\frac{\delta_{2}}{r_{2}}N_{\text{Ez}} \end{array}\right.$$

Where, Q_1_ is traction force generated by motor 1, N; F_Ax_, N_Ay_, N_Az_ are forces acting on the x-axis, y-axis, and z-axis directions between the walking wheels of the rack and the track at point A, N; F_Bx_, N_By_, N_Bz_ are forces acting on the x-axis, y-axis, and z-axis directions between the walking wheels of the rack and the track at point B, N; F_Cx_, N_Cy_, N_Cz_ are forces acting on the x-axis, y-axis, and z-axis directions between the walking wheels of the rack and the track at point C, N; F_Dx_, N_Dy_, N_Dz_ are forces acting on the x-axis, y-axis, and z-axis directions between the walking wheels of the rack and the track at point D, N; F_Ex_ is rolling friction between the walking wheel of the push-pull rod and the ground, N; N_Ez_ is force between the push-pull rod support wheel and the contact surface, N; m_1_ is mass of the body frame, kg; m_2_ is mass of the spray bracket transfer platform, kg; m_3_ is mass of the push-pull rod, kg; l_1_ is distance between point O and point E on the push-pull rod, m; l_2_ is distance between two sets of contact rollers, m; l_3_ is half the distance between the front and rear walking wheels, m; f_1_ is kinetic friction factor between the walking wheel of the body frame and the rack; δ_1_ is coefficient of rolling resistance between the walking wheel of the body frame and the rack, mm; r_1_ is radius of the body frame’s walking wheels, mm; δ_2_ is coefficient of rolling resistance between the walking wheel of the push-pull rod and the ground, mm; r_2_ is radius of the push-pull rod’s walking wheels, mm.

The walking wheel is engineered with a T-shaped single-side structure. It adheres firmly to the outer side of the track. This configuration is engineered to counterbalance the asymmetrical lateral force and the torque generated by the push-pull rod, thereby ensuring the stability and equilibrium of the nutrient solution supply device during operation.

In order to facilitate movement of the push-pull rod relative to the body frame while simultaneously restricting its position, four rotating contact rollers are installed at the midpoint of the body frame. In the event of the body frame being in a state of stationarity, whilst the push-pull rod moves along its axis, the contact rollers rotate synchronously. Conversely, when the body frame is in motion, the push-pull rod is driven by the body frame to move along its vertical axis. The contact rollers are engineered to restrict the push-pull rod, thereby ensuring its movement is confined to the vertical axis, in synchrony with the body frame. This configuration is designed to prevent undesirable deviations or vibrations, thereby ensuring the accuracy and reliability of the system’s operation.

**Figure 6:**
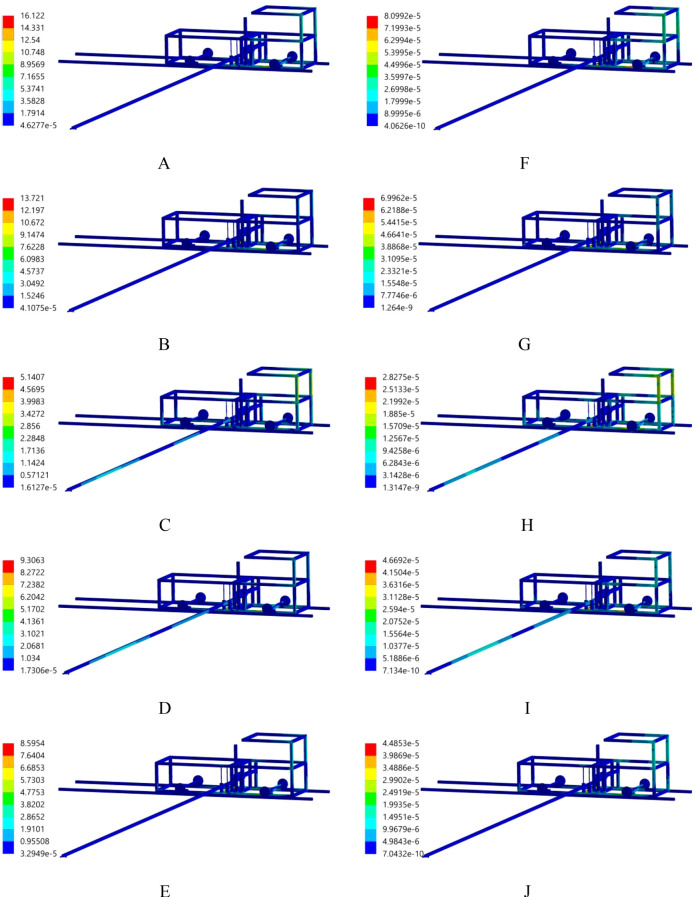
Equivalent stress and strain distribution. **(A)** stress, 0.2s; **(B)** stress, 0.4s; **(C)** stress, 0.6s; **(D)** stress, 0.8s; **(E)** stress, 1.0s; **(F)** strain, 0.2s; **(G)** strain, 0.4s; **(H)** strain, 0.6s; **(I)** strain, 0.8s; **(J)** strain, 1.0s.

The transient dynamics analysis of the body frame driving the movement of a 6-metre-long push-pull rod is conducted using ANSYS Workbench 2025 R1 (student) software. When the body frame is set to move at a speed of 0.15 m/s, the distributions of the equivalent stress and equivalent strain of the body frame driving the push-pull rod are shown in **Figure 6**. It is evident from the distributions that during the movement process, the maximum stress value of the body frame is 16.122 MPa, which is far lower than the allowable stress of the material. The stress distribution is found to be uniform, with no discernible stress concentration areas observed. The strain of all parts of the body frame is found to be minimal, with a value of only 8.099×10–^5^ mm. This finding indicates that, at this particular velocity, the body frame functions in a stable manner and the structural design parameters are in alignment with the operational requirements, thereby ensuring the reliability and stability of the nutrient solution supply device during actual operation.

#### 2.3.4 Spray bracket and push-pull rod

**Figure 7:**
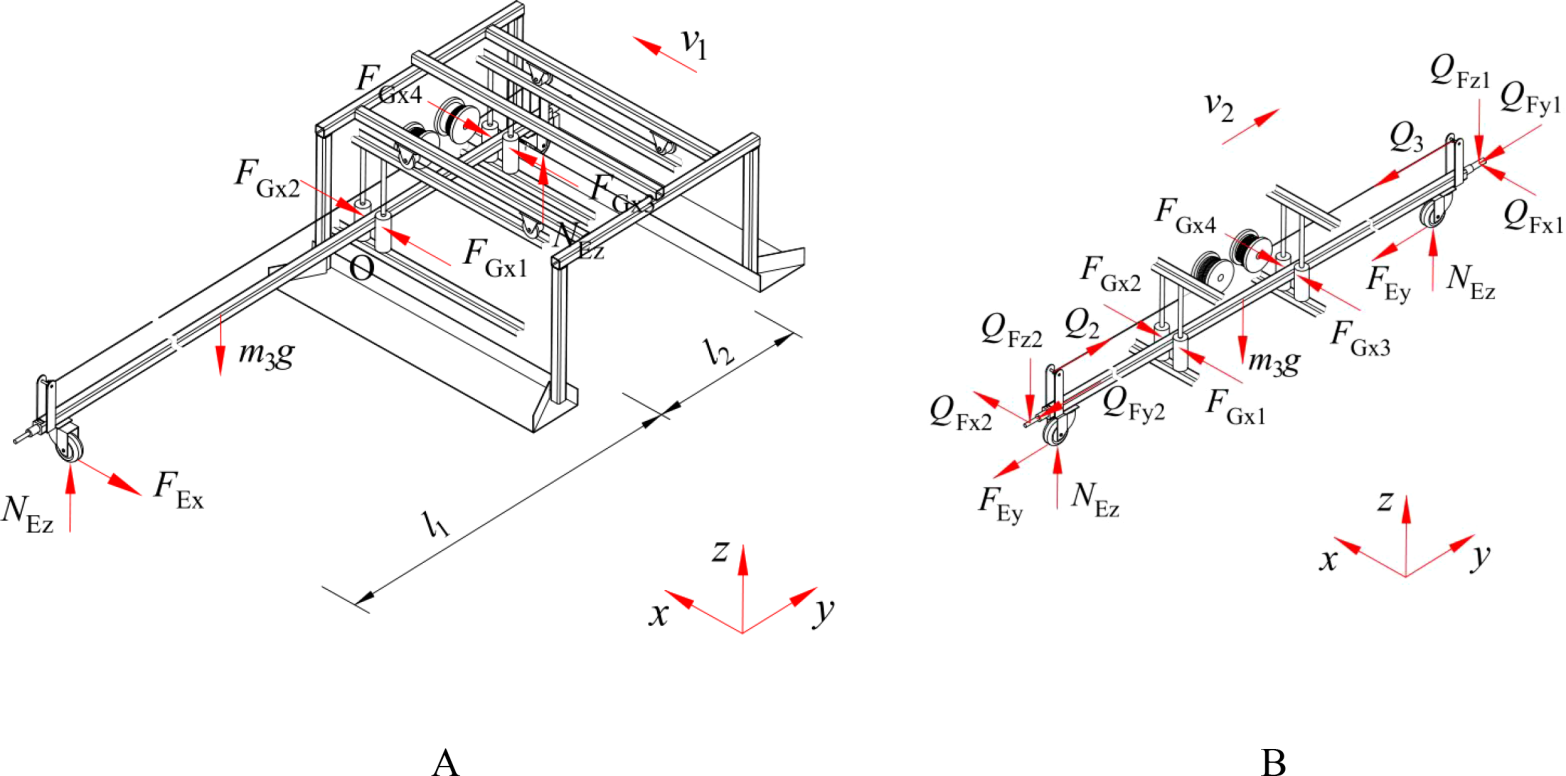
Force-bearing condition of the push-pull rod. **(A)** Movement of the push-pull rod driven by the body frame; **(B)** Movement of the push-pull rod along its axial direction.

A wheel is installed at each extremity of the push-pull rod. Furthermore, four contact rollers are utilized to facilitate the connection between the push-pull rod and the body frame through high-pair contact. When the push-pull rod is driven to move by the body frame, ignoring the acting force of the spray bracket on the push-pull rod and the rolling friction force of the contact rollers on the push-pull rod, its force-bearing condition is shown in **Figure 7A**, and the mechanical relationship is expressed by Equation 4.


(4) $$\left\{ \begin{array}{l} F_{\text{Gx}1}-F_{\text{Gx}2}+F_{\text{Gx}3}-F_{\text{Gx}4}-F_{\text{Ex}}=0\\ 2N_{\text{Ez}}-m_{3}g=0\\ (F_{\text{Gx}4}-F_{\text{Gx}3})\cdot l_{2}-F_{\text{Ex}}\cdot l_{1}=0\\ F_{\text{Ex}}=\frac{\delta_{2}}{r_{2}}N_{\text{Ez}} \end{array}\right.$$

Where, F_Gx1_, F_Gx2_, F_Gx3_, F_Gx4_ are forces exerted by the two sets of contact rollers on the push-pull rod, N.

**Figure 8:**
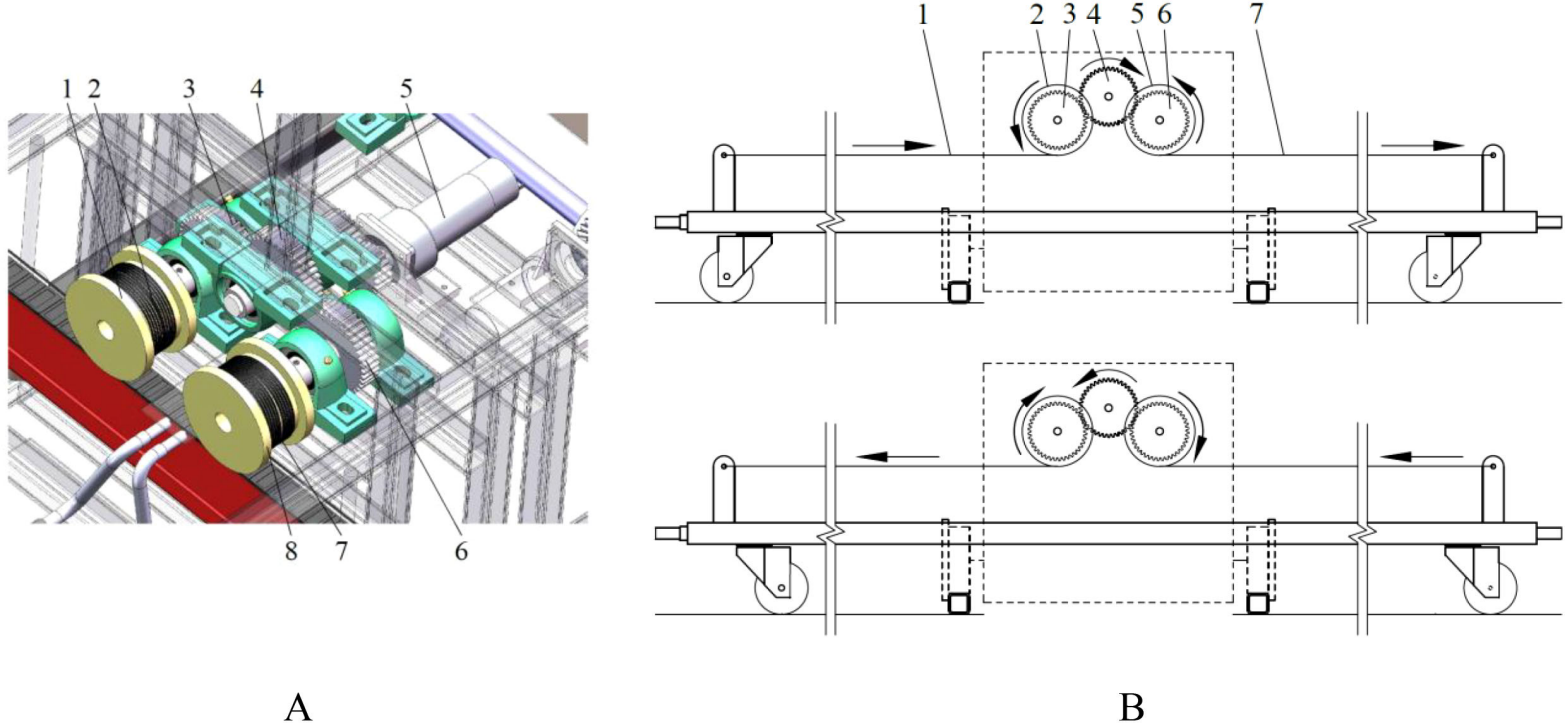
Movement process of the push-pull rod. **(A)** driving component assembly of the push-pull rod. 1.winding roller 1 2. pulling wire 1 3. gear 1 4. gear 3 5. drive motor for the push-pull rod 6. gear 2 7. winding roller 2 8. pulling wire 2; **(B)** movement process in the direction of the axis of the push-pull rod. **(a)** push-pull rod moves to the left. 1. pulling wire 1 2. winding roller 1 3. gear 1 4. gear 3 5. winding roller 2 6. gear 2 7. pulling wire 2; **(b)** push-pull rod moves to the right.


The driving component assembly of the push-pull rod is shown in **Figure 8A**. The movement process of the push-pull rod along its axial direction is presented in **Figure 8B**. When the drive motor for the push-pull rod is set to rotate clockwise, the gear 3 connected to it also rotates accordingly. Consequently, gear 1 is driven to rotate in the same direction as gear 2. Within this transmission process, the winding roller 1, driven by gear 1, undergoes a take-up process, while the winding roller 2, driven by gear 2, undergoes a pay-off process. The combined effect of these processes, namely the take-up and pay-off actions, results in the movement of the push-pull rod to the right. Conversely, when the drive motor for the push-pull rod rotates in the counterclockwise direction, the movement directions of each gear and the winding rollers are opposite to the abovementioned process, and the winding rollers drive the push-pull rod to move to the left. The movement speed of the push-pull rod is calculated as in Equation 5.

(5) $$v_{2}=\frac{\pi d_{2}n_{2}}{6\times 10^{4}i_{2}}$$

Where, *v*_2_ is the moving speed of the push-pull rod, m/s; *d*_2_ is the diameter of the winding roller, mm; *n*_2_ is the rotational speed of the drive motor for the push-pull rod, r/min; *i*_2_ is the transmission ratio of the master-slave wheel.

When the push-pull rod moves along its axis, ignoring the rolling friction force of the contact rollers on the push-pull rod, the force-bearing condition is shown in **Figure 7B**, and the mechanical relationship is expressed by Equation 6.


(6) $$\left\{ \begin{array}{l} F_{Gx1}-F_{Gx2}+F_{Gx3}-F_{Gx4}+Q_{Fx1}+Q_{Fx2}=0\\ Q_{2}-Q_{3}-2F_{\text{Ey}}-Q_{Fy1}-Q_{Fy2}=0\\ Q_{Fz1}+Q_{Fz2}+m_{3}g-2N_{\text{Ez}}=0\\ F_{\text{Ey}}=\frac{\delta_{2}}{r_{2}}N_{\text{Ez}} \end{array}\right.$$

Where, Q_Fx1_, Q_Fy1_, Q_Fz1_ are forces exerted by the push-pull rod to push the spray bracket on the x-axis, y-axis, and z-axis, N; Q_Fx2_, Q_Fy2_, Q_Fz2_ are forces exerted by the push-pull rod to drag the spray bracket along the x-axis, y-axis, and z-axis, N; Q_2_ is tensile force in the rope during winding by the rope winding roller, N; Q_3_ is tensile force in the rope during unwinding by the rope winding roller, N.

**Figure 9:**
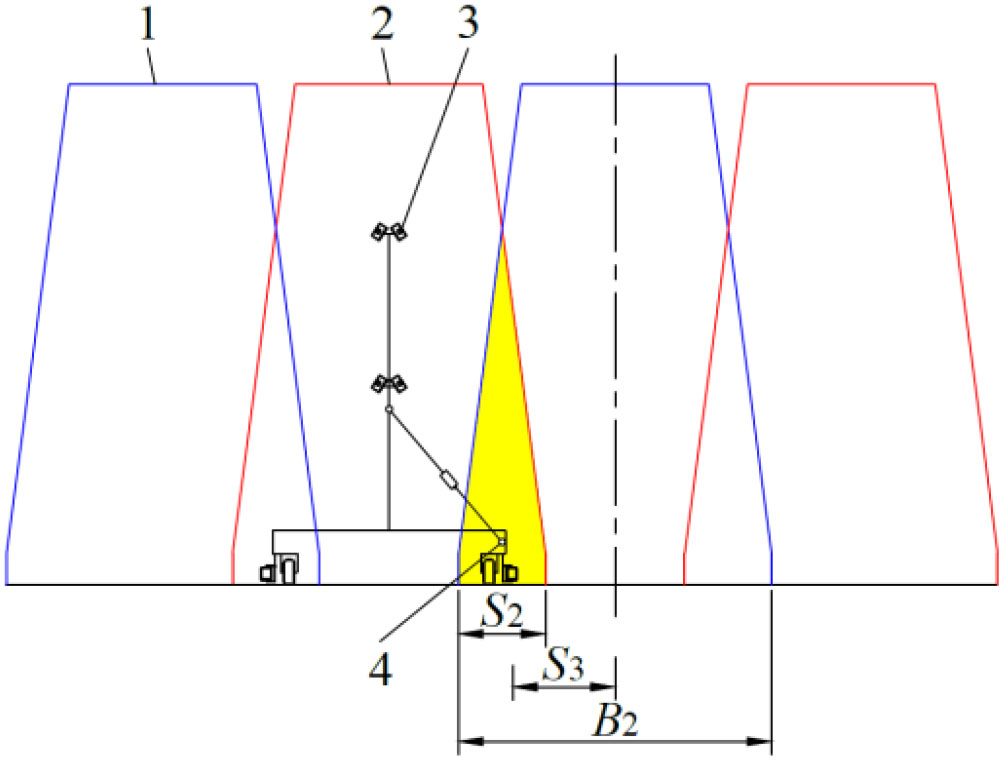
Longitudinal direction projection of the cultivation beds located on both sides of the nutrient solution supply pool. 1. cultivation bed located on one side of the nutrient solution supply pool 2. cultivation bed located on the other side of the nutrient solution supply pool 3. spray bracket 4. projection of the axis of the push-pull rod.


The longitudinal direction projection of the cultivation beds on both sides of the nutrient solution supply pool is shown in **Figure 9**. It is imperative to consider the positioning of the push-pull rod within the cultivation beds, which are staggered on both sides of the nutrient solution supply pool. The distance between the push-pull rod’s axis and the longitudinal symmetric plane of the cultivation bed is determined as in Equation 7.

(7) $$S_{3}=\frac{B_{2}-S_{2}}{2}$$

Where, *S*_3_ is the distance between the axis of the push-pull rod and the longitudinal symmetric plane of the cultivation bed, m; *B*_2_ is the length of the lower base of the cultivation bed cross-section, m; *S*_2_ is the maximum horizontal length of the overlapping area of the longitudinal projections of the cultivation beds on both sides of the nutrient solution supply pool, m.

**Figure 10:**
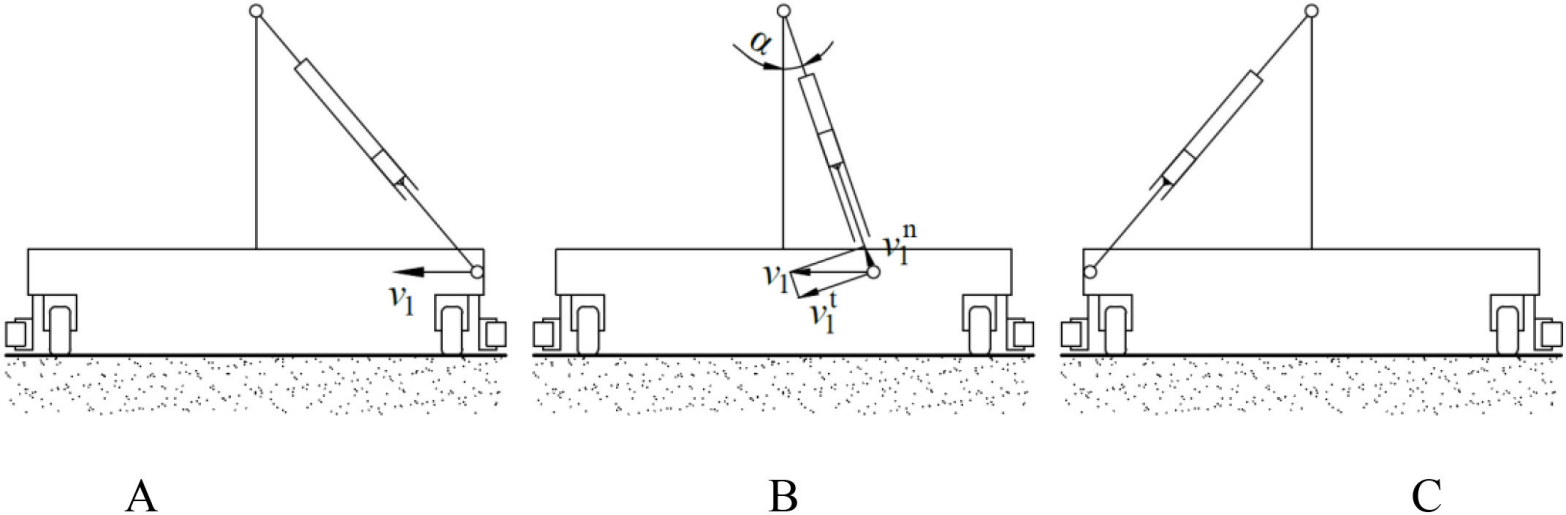
Movement process in the direction of the perpendicular to the axis of the push-pull rod. **(A)** initial position; **(B)** any intermediate position between initial position and final position; **(C)** final position.


The structural configuration of the spray bracket is illustrated in **Figure 2**. A revolute pair connection form is adopted between the push-pull rod and the spray bracket. The movement state of the connection point is adjusted by the spray bracket via a sliding-block structure that incorporates a guide rail. When the push-pull rod is moved by the body frame, the spray bracket remains stationary, while the connecting rod of the spray bracket moves together with the push-pull rod, as demonstrated in **Figure 10**. The speed of the connecting rod of the spray bracket is expressed by Equation 8.

(8) $$\left\{ \begin{array}{l} \omega =\frac{v_{1}\cos\alpha }{r_{\text{f}}}\\ v_{1}^{n}=v_{1}\sin\alpha \end{array}\right.$$

Where, *ω* is rotation angular speed of the connecting rod of the spray bracket, rad/s; *α* is angle between the connecting rod of the spray bracket and the vertical direction; *r*_f_ is distance between the connection point and the rotation point, m; $v_{1}^{n}$ is linear speed of the connection rod moving along the guide rail, m/s.

**Figure 11:**
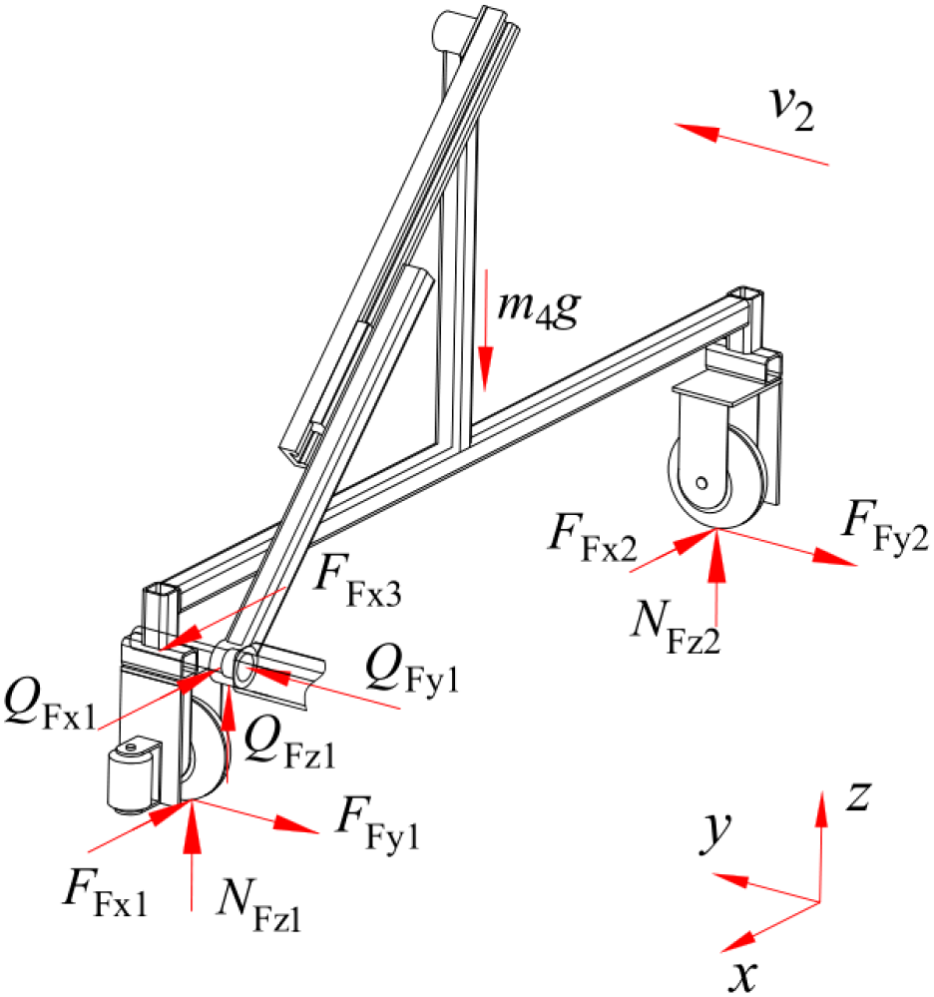
Force-bearing condition of the spray bracket.


In the event of movement of the push-pull rod along its axial direction, the speed of movement of the spray bracket is consistent with that of the push-pull rod. This is the operational process of nutrient solution supply. When the spray bracket is in motion under the push of the push-pull rod, its force-bearing condition is shown in **Figure 11**, and the mechanical relationship is expressed by Equation 9.

(9) $$\left\{ \begin{array}{l} F_{Fx1}+F_{Fx2}-F_{Fx3}+Q_{Fx1}=0\\ Q_{Fy1}-F_{Fy1}-F_{Fy2}=0\\ N_{Fz1}+N_{Fz2}+Q_{Fz1}-m_{4}g=0\\ F_{Fy1}=\frac{\delta_{3}}{r_{3}}N_{Fz1}\\ F_{Fy2}=\frac{\delta_{3}}{r_{3}}N_{Fz2} \end{array}\right.$$

Where, F_Fx1_, F_Fy1_, N_Fz1_ are forces in the x-axis, y-axis, and z-axis directions between the walking wheels of the spray bracket on the side close to the push-pull rod and the ground, N; F_Fx2_, F_Fy2_, N_Fz2_ are forces in the x-axis, y-axis, and z-axis directions between the walking wheels of the spray bracket on the side far from the push-pull rod and the ground, N; Q_Fx1_, Q_Fy1_, Q_Fz1_ are forces in the x-axis, y-axis, and z-axis directions at the revolute joint between the push-pull rod and the spray bracket, N; F_Fx3_ is contact forces between the push-pull rod and the spray bracket, N; m_4_ is the mass of the spray bracket, kg; δ_3_ is coefficient of rolling resistance between the walking wheel of the spray bracket and the ground, mm; r_3_ is radius of the spray bracket’s walking wheels, mm.

**Figure 12:**
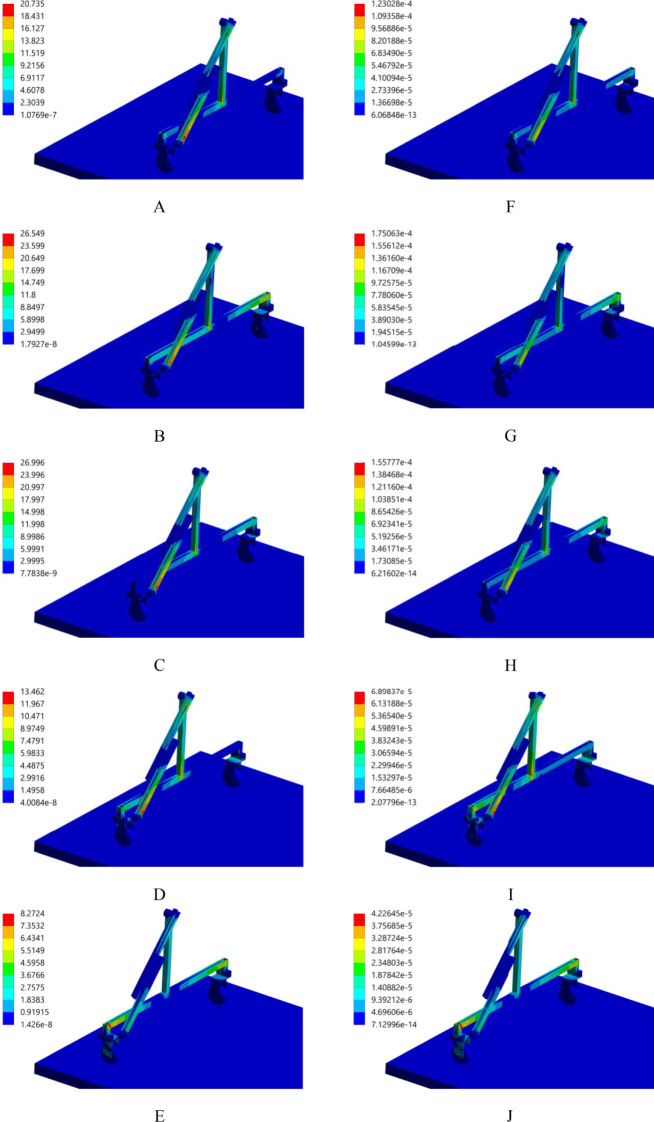
Equivalent stress and strain distribution. **(A)** stress, 0.2s; **(B)** stress, 0.4s; **(C)** stress, 0.6s; **(D)** stress, 0.8s; **(E)** stress, 1.0s; **(F)** strain, 0.2s; **(G)** strain, 0.4s; **(H)** strain, 0.6s; **(I)** strain, 0.8s; **(J)** strain, 1.0s.

The transient dynamics analysis of the movement of the spray bracket is conducted using ANSYS Workbench 2025 R1 (student) software. When the moving speed of the spray bracket is 0.4 m/s, the distributions of the equivalent stress and equivalent strain of the spray bracket are shown in **Figure 12**. It is evident from the distributions that during movement, the maximum stress value of the spray bracket is 26.996 MPa, which is significantly lower than the allowable stress of the material. The stability of the working states at the connection points between the push-pull rod and the revolute pair of the spray bracket, as well as at the slider-guide rail, is evident, with no obvious stress-concentration areas appearing. The strain values of all parts of the spray bracket are very small, with the maximum being 1.751×10^-4^ mm, indicating that the structural design of this spray bracket is reasonable and the operation is stable.

In the structural design of the spray bracket, a mechanical connection is established with the spray bracket through the connection points at the ends of the push-pull rod. Additionally, contact points with the frame of the spray bracket are configured. This configuration is designed to effectively prevent the spray bracket from shifting towards the side of the push-pull rod during operation. This ensures that the spray bracket does not deviate towards the side of the push-pull rod during work, thereby guaranteeing the stability and reliability of the nutrient solution supply device. The installation of protection wheels on both sides of the spray bracket is pivotal in preventing deviation towards the cultivation bed during movement, thus averting friction and collision phenomena. The width range of the spray bracket is determined by Equation 10.


(10) $$2S_{1}<L_{2}<B_{2}$$

Where, *L*_2_ is the length of the spray bracket, m.

The height of the spray bracket must be determined in accordance with the dimensions and configuration of the cultivation bed, as well as the number and arrangement of the requisite spray nozzles.

#### 2.3.5 Spray bracket transfer platform

The spray bracket transfer platform, as depicted in **Figure 2**, is driven by a rack and pinion drive, which moves at a speed of movement is calculated by Equation 11.


(11) $$v_{3}=\frac{\pi d_{3}n_{3}}{6\times 10^{4}}$$

Where, *v*_3_ is the moving speed of the spray bracket transfer platform, m/s; *d*_3_ is the diameter of the gear indexing circle, mm; *n*_3_ is the rotational speed of the drive motor for the spray bracket transfer platform, r/min.

**Figure 13:**
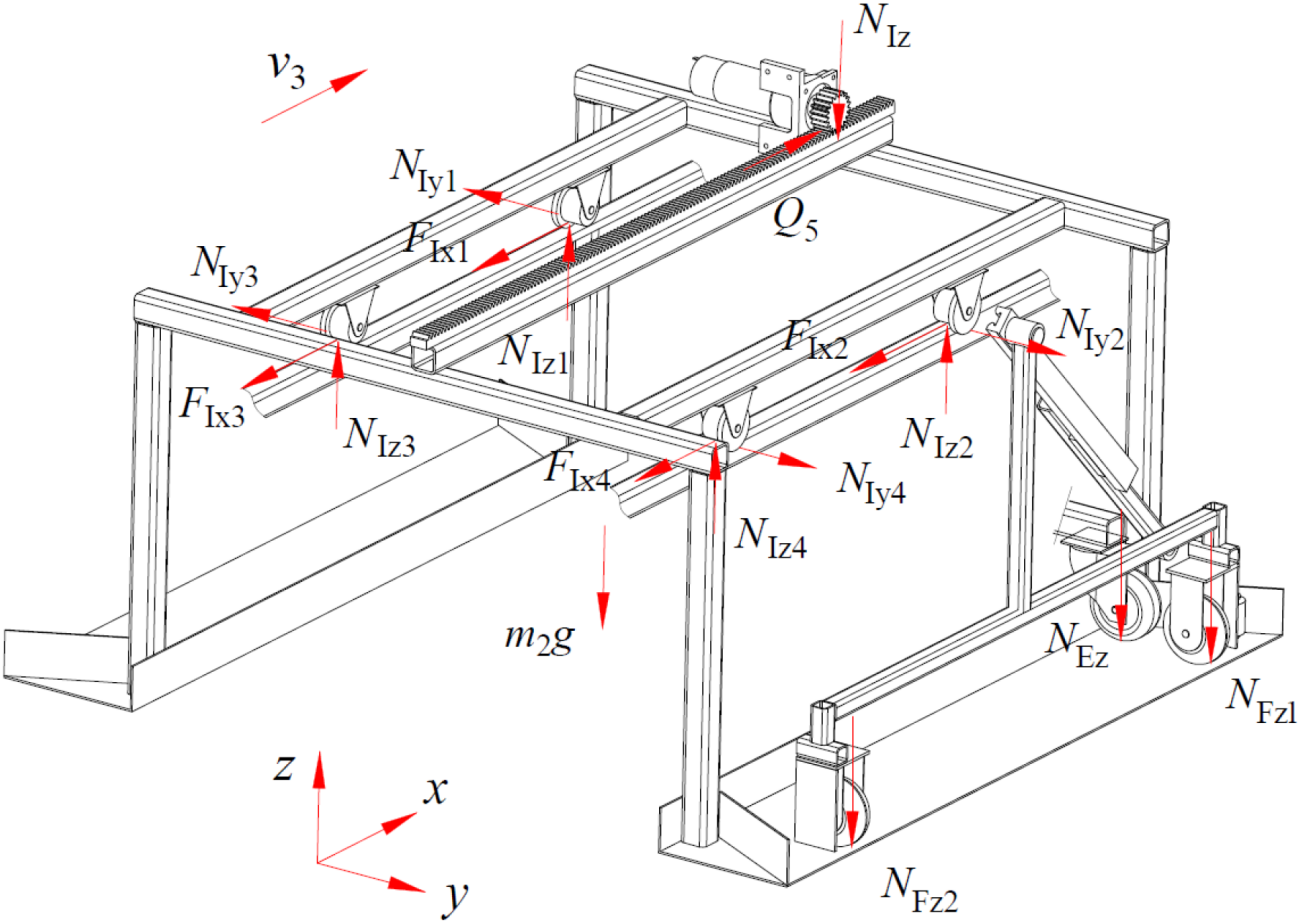
Force-bearing condition of the spray bracket transfer platform.


When the spray bracket transfer platform is in motion, its force-bearing condition is shown in **Figure 13**, and the mechanical relationship is expressed by Equation 12.

(12) $$\left\{ \begin{array}{l} Q_{5}-(F_{Ix1}+F_{Ix2}+F_{Ix3}+F_{Ix4})=0\\ N_{Iy1}-N_{Iy2}+N_{Iy3}-N_{Ix4}=0\\ N_{Iz1}+N_{Iz2}+N_{Iz3}+N_{Iz4}-m_{2}g-N_{\text{Ez}}-N_{\text{Iz}}-N_{Fz1}-N_{Fz2}=0\\ F_{Ix1}=f_{2}N_{Iy1}+\frac{\delta_{4}}{r_{4}}N_{Iz1}\\ F_{Ix2}=f_{2}N_{Iy2}+\frac{\delta_{4}}{r_{4}}N_{Iz2}\\ F_{Ix3}=f_{2}N_{Iy3}+\frac{\delta_{4}}{r_{4}}N_{Iz3}\\ F_{Ix4}=f_{2}N_{Iy4}+\frac{\delta_{4}}{r_{4}}N_{Iz4} \end{array}\right.$$

Where, Q_5_ is traction force generated by motor 2, N; F_Ix1_, N_Iy1_, N_Iz1_; F_Ix2_, N_Iy2_, N_Iz2_; F_Ix3_, N_Iy3_, N_Iz3_; F_Ix4_, N_Iy4_, N_Iz4_ are forces in the x-axis, y-axis, and z-axis directions between each of the four walking wheels of the spray bracket transfer platform and the body frame, N; N_Iz_ is pressure exerted by the motor 2 on the spray bracket transfer platform, N; f_2_ is kinetic friction factor between the walking wheel of the spray bracket transfer platform and the body frame; δ_4_ is coefficient of rolling resistance between the walking wheel of the spray bracket transfer platform and the body frame, mm; r_4_ is radius of the spray bracket transfer platform’s walking wheels, mm.

The function of the spray bracket transfer platform is to facilitate the transportation of spray brackets between different cultivation beds. Its length is determined by Equation 13.


(13) $$L_{3}=1.1L_{2}$$

In the event of the push-pull rod exerting a force on the spray bracket, causing it to move onto the spray bracket transfer platform, the baffle on the latter is able to recalibrate the relative position of the spray bracket and the cultivation bed, thus enabling it to return to the preset position. This realignment operation is performed each time the push-pull rod retracts the spray bracket to the spray bracket transfer platform. This ensures that the spray bracket consistently maintains the optimal relative position with the cultivation bed across different operational phases, thereby guaranteeing efficient and stable operation.

#### 2.3.6 Nutrient solution spraying system

**Figure 14:**
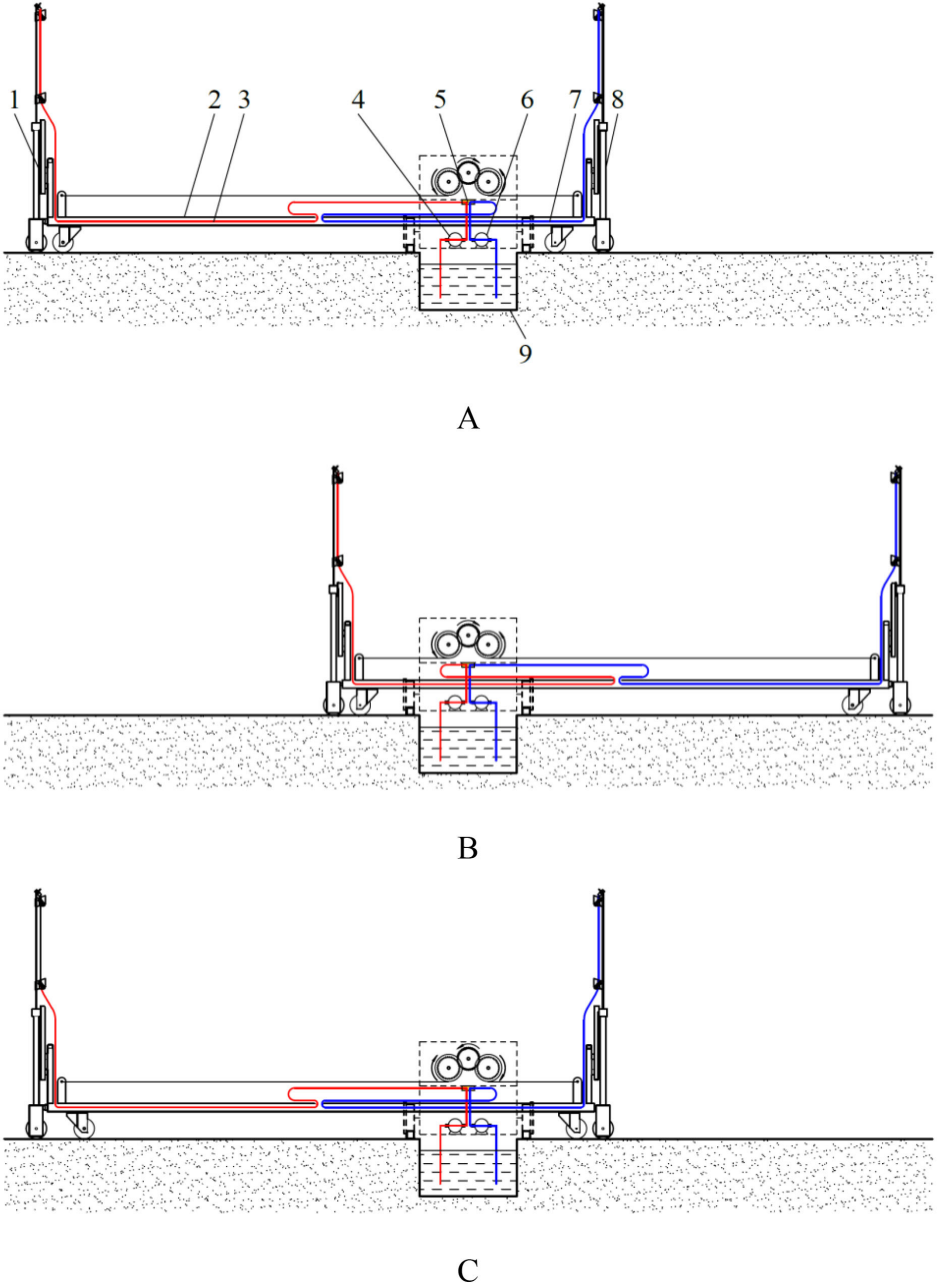
Nutrient solution spraying system in operation. **(A)** initial state. 1. spray bracket 1 2. push-pull rod 3. transmission pipeline of nutrient solution 1 4. water pump 1 5. fixing point at the body frame 6. water pump 2 7. transmission pipeline of nutrient solution 2 8. spray bracket 2 9. nutrient solution supply pool; **(B)** final state; **(C)** return to the initial state.

The nutrient solution spraying system is mainly composed of water pumps, transmission pipelines of nutrient solution, and spray nozzles, as illustrated in **Figure 14**. The system is equipped with two identical subsystems, the function of which is to supply nutrient solution to the cultivation beds in the areas on both sides of the supply pool, ensuring comprehensive and balanced nutrient solution supply. The transmission pipelines of the nutrient solution originate at the fixing point on the body frame and enter the interior of the drag chain, which is set between the push-pull rod and the fixing point on the body frame. An opening is made in the middle of the push-pull rod. After passing through this opening, the transmission pipelines of nutrient solution enter the lumen of the push-pull rod and pass out from both ends of it, finally reaching the spray bracket smoothly. The determining the power of water pumps and the number and distribution of spray nozzles necessitates a meticulous consideration of multiple factors. The size and shape of the cultivation bed cross-section, as well as the movement speed of the spray bracket, are among the factors that must be taken into consideration. Through the precise coordination of these parameters, it is ensured that the nutrient solution spraying system can adapt with flexibility to the nutrient solution supply requirements in various cultivation environments. This, in turn, provides a reliable guarantee for the stable operation of the nutrient solution supply device.

### 2.4 Experimental design

#### 2.4.1 Experimental equipment, instruments and materials

The equipment, instruments, and materials utilized in this experiment primarily comprise a nutrient solution supply device, test frames, iron wires, a water box, a water bucket, chalk, double-sided adhesive tape, water-sensitive paper, sealed bags, a tachometer, a meter stick, a tape measure, and nutrient solution.

#### 2.4.2 Experimental factors and evaluation indicator

In this experiment, the flow rate of the spray nozzles, the moving speed of the spray nozzles, and the vertical height from the test points on the legs of the trapezoidal cross-section of the cultivation bed to the ground are selected as the experimental factors. The nutrient solution coverage rate is utilized as the evaluation indicator, given its capacity to effectively measure the working effectiveness of the device. The formula for calculating nutrient solution coverage rate is calculated using Equation 14.


(14) $$M=\frac{R_{1}}{R}\times 100\%$$

Where, *M* is the nutrient solution coverage rate, %; *R*_1_ is the area of the water-sensitive paper covered by nutrient solution, mm^2^; *R* is the area of the water-sensitive paper, mm^2^.

#### 2.4.3 Experimental scheme

The study employs the Box-Behnken experimental design, incorporating three factors and three levels. The experimental factors and codes are shown in **Table 3**, and the experimental scheme is illustrated in **Table 4**.

**Table 3:** Experimental factors and codes.

Code value	Flow rate of the spray nozzles A (L/min)	Moving speed of the spray nozzles B (m/s)	Vertical height from the test points on the legs of the trapezoidal cross-section of the cultivation bed to the ground C (m)
-1	2.4	0.3	0.1
0	2.7	0.35	0.75
1	3	0.4	1.4

**Table 4:** Experimental scheme and results.

No.	Experimental factors			The coverage rate of the nutrient solution *Y* (%)
	*X*_1_	*X*_2_	*X*_3_	
1	-1	-1	0	87.49
2	1	-1	0	100
3	-1	1	0	81.48
4	1	1	0	87.34
5	-1	0	-1	85.7
6	1	0	-1	94.52
7	-1	0	1	85.07
8	1	0	1	95.84
9	0	-1	-1	91.41
10	0	1	-1	80.28
11	0	-1	1	91.58
12	0	1	1	78.47
13	0	0	0	89.22
14	0	0	0	91.27
15	0	0	0	88.67
16	0	0	0	90.85
17	0	0	0	88.62

#### 2.4.4 Experiment methods

**Figure 15:**
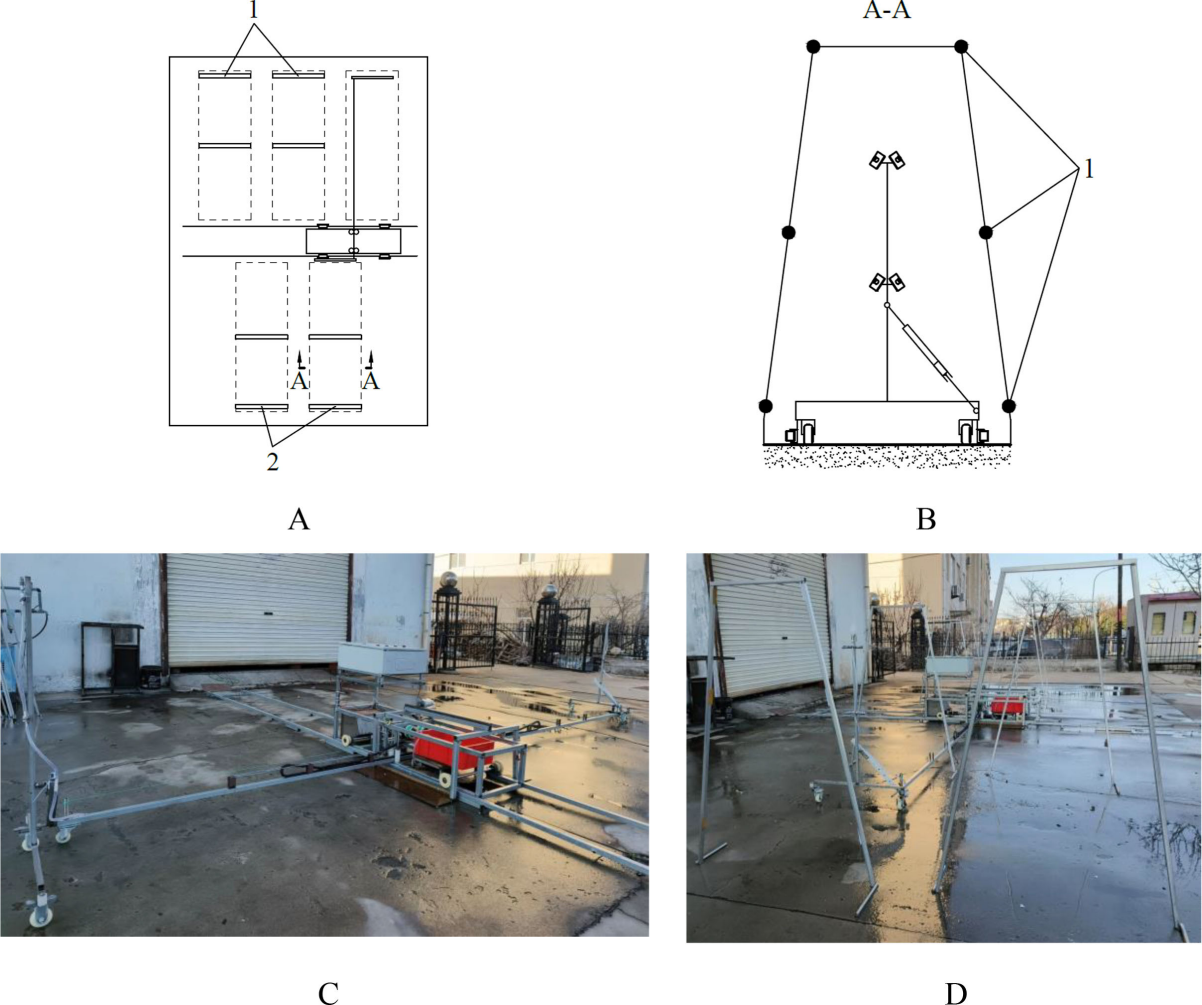
Experimental site’s planar layout and experimental device. **(A)** test area. 1. test positions on one side of the nutrient solution supply pool 2. test positions on the other side of the nutrient solution supply pool; **(B)** trapezoidal cross-section of the cultivation bed. 1. test points on the legs of the trapezoidal cross-section of the test frame; **(C)** experimental device, **(D)** experimental device in operation.

The experimental working area of the nutrient solution supply device was set up on the flat ground. The experimental site’s planar layout and the operational state of the experimental device were illustrated in **Figure 15**. On both sides of the nutrient solution supply device’s track, two cultivation bed distribution areas were demarcated respectively as the designated test areas. Within each designated test area, two test positions were established at the midpoint and at the far end of the cultivation bed distribution area, respectively, with a test frame located at each test position. The test frame was constructed as an isosceles trapezoid frame, which was used to imitate the trapezoidal cross-section of the cultivation bed. Test points were set at the positions where the vertical height from the two waistlines of the trapezoid on the test frame to the ground was 0.1m, 0.75m, and 1.4m.

Prior to the execution of the test, in accordance with the stipulated experimental scheme, the water-sensitive papers were affixed at the designated test points and numbered. Subsequent to the completion of one test, the water-sensitive paper was meticulously detached from each test point in succession, and then positioned on the paper surface. Following the complete drying of the water-sensitive paper and the stabilization of the water marks, which were no longer subject to change, the paper was then correctly stored in a sealed bag.

Each experimental scheme was conducted in eight replicates across two cycles, with four identical cultivation beds tested per cycle, two sampling points set per bed, and two measurements collected at the same height for each sampling point. The water-sensitive papers were scanned into digital images, then converted into the DXF format using R2V 5.5 software. The area that was sprayed by the nutrient solution was then measured using AutoCAD 2024 software. Nutrient solution coverage rate was calculated on the water-sensitive paper, and the average value of the nutrient solution coverage rate at the same height position was also calculated.

## 3 Results

The experimental results are displayed in **Table 4**. The variance analysis of the experimental results is conducted using Design-Expert 13.0 software, and the analysis results are presented in **Table 5**. The regression model is highly significant (P < 0.01), while the lack of fit is not significant (P > 0.05). This indicates that the regression model has a good goodness of fit and is well-fitted.

**Table 5:** Variance analysis.

Source	Sum of squares	df	Mean square	F-value	P-value
Model	481.87	9	53.54	34.12	< 0.0001**
*X*_1_	180.12	1	180.12	114.78	< 0.0001**
*X*_2_	230.16	1	230.16	146.67	< 0.0001**
*X*_3_	0.1128	1	0.1128	0.0719	0.7963
*X*_1_ *X*_2_	11.06	1	11.06	7.05	0.0327*
*X*_1_ *X*_3_	0.9506	1	0.9506	0.6058	0.4619
*X*_2_ *X*_3_	0.9801	1	0.9801	0.6246	0.4553
*X*_1_²	18.56	1	18.56	11.83	0.0109*
*X*_2_²	31.80	1	31.80	20.26	0.0028**
*X*_3_²	10.02	1	10.02	6.39	0.0394*
Residual	10.98	7	1.57		
Lack of Fit	4.74	3	1.58	1.01	0.4745
Pure Error	6.24	4	1.56		
Cor Total	492.85	16			

** represents highly significant (p< 0.01), * represents significant (p< 0.05).

The fit statistics include an R^2^ value of 0.9777, an Adjusted R^2^ of 0.9491, a Standard Deviation (Std. Dev.) of 1.25, and a Coefficient of Variation (C.V. %) of 1.41. The regression model demonstrates a high degree of explanatory power, as evidenced by the relatively high values of the coefficient of determination (R^2^ = 0.9777) and the adjusted coefficient of determination (adjusted R^2^ = 0.9491). The relatively low Std. Dev. and C.V. % further demonstrate the stability and reliability of the experimental results. The regression model demonstrates a highly significant influence of *X*_1_, *X*_2_, and *X*_2_^2^ on the nutrient solution coverage rate (P < 0.01), and a significant influence of *X*_1_ *X*_2_, *X*_1_^2^, and *X*_3_^2^ on the nutrient solution coverage rate (P < 0.05). Elimination of terms with negligible impact on the nutrient solution coverage rate results in the following regression model is obtained, as shown in Equation 15.


(15) $$Y=89.73+4.75X_{1}-5.36X_{2}-1.66X_{1}X_{2}+2.1X_{1}{\;}^{2}-2.75X_{2}{\;}^{2}-1.54X_{3}{\;}^{2}$$

**Figure 16:**
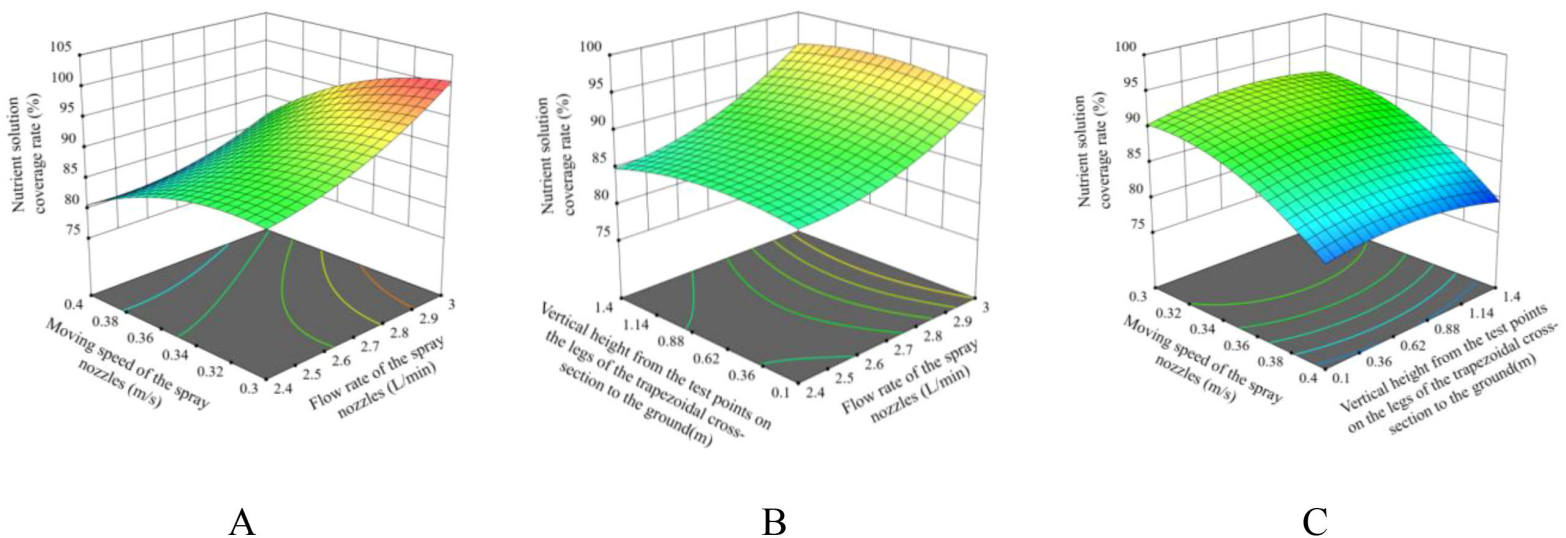
The response surfaces of the effects of two factors on the nutrient solution coverage rate. **(A)** AB; **(B)** AC; **(C)** BC.

The response surfaces illustrating the effects of two factors on the nutrient solution coverage rate are depicted in **Figure 16**. As can be observed from the figure, with the increase in the flow rate of the spray nozzles, the nutrient solution coverage rate shows an upward trend. As the moving speed increases, the nutrient solution coverage rate exhibits a decreasing trend. As the vertical height from the test points on the legs of the trapezoidal cross-section of the cultivation bed to the ground increases, the nutrient solution coverage rate first rises and then declines.

The regression model of the nutrient solution coverage rate reflects the variation law of the nutrient solution coverage rate of each point on the legs of the trapezoidal cross-section of the cultivation bed under different flow rates of the spray nozzles and moving speeds of the spray nozzles of the device. The regression model indicates that, under equivalent operating conditions, when X3 = -1 or 1, the nutrient solution coverage rate at the corresponding height position is minimal. Consequently, if the nutrient solution coverage rate at this position on the legs of the trapezoidal cross-section of the cultivation bed can be ensured to reach the specified range, then the nutrient solution supply requirements can definitely be met simultaneously at other positions. It is crucial to note that, under the condition that the flow rate of the spray nozzles does not compromise root integrity, and within the constraints imposed by the boundary conditions, priority should be accorded to ensuring that the spray nozzles operate at a relatively high velocity. Concurrently, by implementing a flexible adjustment of the flow rate of the spray nozzles, the nutrient solution coverage rate can be elevated to exceed the prescribed standard. The optimization objective function and constraint conditions are established as shown in Equation 16.


(16) $$\left\{ \begin{array}{l} \max Y(X_{1},X_{2},X_{3})\geq 90\\ s.t.\{\begin{array}{l} -1\leq X_{1}\leq 1\\ -1\leq X_{2}\leq 1\\ X_{3}=-1\text{ or }1 \end{array} \end{array}\right.$$

It can thus be concluded that when the flow rate of the spray nozzles is set at 3 L/min, the moving speed of the spray nozzles is 0.38 m/s, and the vertical height from the test points on the legs of the trapezoidal cross-section of the cultivation bed to the ground is 0.1 m and 1.4 m, the nutrient solution coverage rates are 90% and 90.17% respectively. In this working condition, it can be ensured that the nutrient solution coverage rate of each point on the legs of the trapezoidal cross-section of the cultivation bed is greater than 90%.

The optimization results indicate that the flow rate of the spray nozzles is 3 L/min, and the moving speed of the spray nozzles is 0.38 m/s. The nutrient solution coverage rate tests were carried out for the positions where the vertical height from the test points on the legs of the trapezoidal cross-section of the cultivation bed to the ground was 0.1 m, 0.75 m, and 1.4 m, respectively. The procedure of the test was identical to the previous one. The experimental results of the verification process demonstrated that the nutrient solution coverage rates of 90.33%, 100%, and 91.52% at the vertical height from the test points on the legs of the trapezoidal cross-section of the cultivation bed to the ground of 0.1 m, 0.75 m, and 1.4 m, respectively, were obtained. These results indicate that, under these operational conditions, the device meets the performance requirements and is in close alignment with the optimized results.

## 4 Discussion

In this paper, we propose a mobile nutrient solution supply mode for vertical aeroponic cultivation and develop a dedicated mobile nutrient supply device for large-scale production. Currently, no studies have reported on mobile nutrient solution supply systems in aeroponics. All existing aeroponic systems rely on fixed-pipeline nutrient solution supply modes, and previous research findings (e.g., Lawrance et al., 2025; Liu et al., 2025; Qureshi et al., 2025) are derived under fixed-pipeline configurations. Compared with fixed-pipeline systems, the proposed mode eliminates complex underground pipeline networks and high-density supporting spray nozzles, reducing the system’s complexity and cost for large-scale aeroponic production.

Transient dynamic analysis using ANSYS Workbench 2025 R1 (student) showed that the stress and strain distributions of the body frame (0.15 m/s) and spray bracket (0.4 m/s) remained within material safety limits, with no significant stress concentration areas. This confirms the device’s structural robustness and ensures stable operation during dynamic movement, aligning with the unified technical requirements for all mechanical equipment.

The Box-Behnken experimental design identified key parameters affecting nutrient solution coverage: nozzle flow rate (3 L/min), movement speed (0.38 m/s), and vertical height (0.1–1.4 m). The device achieved a coverage rate >90% across the trapezoidal bed’s growth zones, demonstrating its capability to address spatial variability in vertical systems—an often-neglected challenge in traditional aeroponics. Experimental observations suggest that using three nozzle groups while increasing flow rate (without damaging plant roots) could further enhance nutrient supply efficiency, allowing for higher spray bracket movement speeds. Tunio et al. (2021) reported that fixed nozzle spacing caused uneven droplet distribution in lettuce cultivation, whereas the movable design in this study dynamically adapts to different growth stages, improving nutrient uniformity. The nutrient solution spraying system of this device is characterized by its flexibility and diversity in configuration. During the process of nutrient solution supply, a variety of spraying system accessories can be selected according to the diverse needs of the actual application scenarios. The combination of spray nozzles with different flow rates and configurations is a rational choice, and the adaptability of the water pumps to meet the diverse requirements of the application is noteworthy.

With the rapid adoption of artificial intelligence, the Internet of Things, and sensor technologies in fixed-mode aeroponic systems (e.g., Qureshi et al., 2025; Dhanasekar, 2025; Sadek et al., 2024; Elsherbiny et al., 2024; Chowdhury et al., 2021), these advanced technologies can be integrated into mobile nutrient solution supply mode to rapidly enhance its automation and intelligence levels.

## 5 Conclusions

(1) This study presents a vertical aeroponic cultivation paradigm for large-scale production and analyses the operational framework.
(2) The paper designs a dedicated nutrient solution supply device, which encompasses a power system, a control system, a body frame, a spray bracket along with its push-pull rod, a spray bracket transfer platform, etc. The subsequent analysis addresses the working principle, technical parameters and operation rules of the nutrient solution supply device.
(3) Transient dynamics analysis via Ansys Workbench 2025 R1 (student) reveals that the distribution of equivalent stress and equivalent strain generated at 0.15 m/s for the body frame and at 0.4 m/s for the spray bracket remain below material allowable limits, with negligible strain. The device demonstrates stable operation and structural integrity, meeting functional requirements.
(4) This study adopted the Box-Behnken experimental design method, analyzed the results using Design Expert 13.0 software, and established a regression model. Optimization results showed that at a nozzle flow rate of 3 L/min and moving speed of 0.38 m/s, the nutrient solution coverage rates at test points with vertical heights of 0.1 m and 1.4 m (from the trapezoidal cross-section legs to the ground) both exceeded 90%. The verification experiment confirmed that under this optimized combination, the coverage rates at the two test points were 90.33% and 91.52%, respectively, meeting actual application requirements.

## Data Availability

The original contributions presented in the study are included in the article/supplementary material. Further inquiries can be directed to the corresponding author.
